# Boosting ^1^H and ^13^C NMR signals by orders of magnitude on a bench

**DOI:** 10.1126/sciadv.adq3780

**Published:** 2024-12-04

**Authors:** Charlotte Bocquelet, Nathan Rougier, Huu-Nghia Le, Laurent Veyre, Chloe Thieuleux, Roberto Melzi, Armin Purea, Daniel Banks, James G. Kempf, Quentin Stern, Ewoud Vaneeckhaute, Sami Jannin

**Affiliations:** ^1^ Universite Claude Bernard Lyon 1, CNRS, ENS Lyon, CRMN UMR 5082, 69100 Villeurbanne, France.; ^2^ Universite Claude Bernard Lyon 1, Institut de Chimie de Lyon, CP2M UMR 5128 CNRS-UCBL-CPE Lyon, 69616 Villeurbanne, France.; ^3^ Bruker Italia S.r.l., Viale V. Lancetti 43, 20158 Milano, Italy.; ^4^ Bruker BioSpin, 76287 Rheinstetten, Germany.; ^5^ Bruker BioSpin, Billerica, MA 01821, USA.

## Abstract

Sensitivity is often the Achilles’ heel of liquid-state nuclear magnetic resonance (NMR) experiments. This problem is perhaps most pressing at the lowest fields (e.g., 80-MHz ^1^H frequency), with rapidly increasing access to NMR through benchtop systems, but also sometimes for higher-field NMR systems from 300 MHz to 1.2 GHz. Hyperpolarization by dissolution dynamic nuclear polarization (dDNP) can address this sensitivity limitation. However, dDNP implies massive and complex cryogenic and high-field instrumentation, which cannot be installed on the bench. We introduce here a compact helium-free 1-T tabletop polarizer as a simple and low-cost alternative. After freezing and polarizing the frozen analyte solutions at 77 K, we demonstrate ^1^H signal enhancement factors of 100, with rapid 1-s buildup times. The high polarization is subsequently transferred by ^1^H→^13^C cross polarization (CP) to ^13^C spins. Such a simple benchtop polarizer, in combination with hyperpolarizing solid matrices (HYPSOs), may open the way to replenishable hyperpolarization throughout multiple liquid-state NMR experiments.

## Teaser

A compact polarizer is designed to enhance the NMR sensitivity by two orders of magnitude for a pure sample on the bench.

## INTRODUCTION

Hyperpolarization methods provide a way to tackle the low sensitivity inherently associated with nuclear magnetic resonance (NMR) and acquire more intense signals in shorter times. This problem of sensitivity is becoming even more pressing today with the fast-growing democratization of low-field benchtop NMR systems. Among the wide array of hyperpolarization strategies available nowadays (*1*), dissolution dynamic nuclear polarization (dDNP) stands out due to its ability to boost nuclear spin polarization—and therefore sensitivity—of almost any small molecular target with long enough nuclear spin-lattice relaxation in solution (typically >1 s) (*2*, *3*). For this reason, dDNP has been overcoming the Boltzmann limits of numerous solution-state magnetic resonance applications (*4*, *5*) in need for trace analyte detection for over 20 years now, especially advancing biomolecular studies such as metabolomics (*6*–*10*), drug discovery (*11*–*14*), or enzymatic reaction monitoring (*15*).

Although dDNP has evolved into a robust, versatile, and even commercially available hyperpolarization technique, a considerable limitation remains its intrinsically destructive and therefore “single shot” character. Once high electron polarization from unpaired radicals has fueled nearby nuclear spin polarization by means of microwave irradiation in a frozen and glassy state at low temperature (1 to 4 K), dissolution with hot and pressurized solvent is performed to rapidly melt and transfer the sample into a liquid-state NMR spectrometer for detection. During the transfer, the sample is irreversibly diluted and sometimes contaminated with radicals and glassing agents—most commonly glycerol—further contributing to the relatively fast relaxation regime in the liquid state (*16*). Dilution can be easily pinpointed to be the critical factor that translates dDNP into a destructive operation. However, it is also a critical factor for the success of dDNP since it attenuates unwanted paramagnetic relaxation induced by the presence of radicals in the liquid solution while simultaneously lowering the viscosity of the solution. Therefore, dilution overall attenuates important loss of polarization. Afterward, only during the hyperpolarization lifetime (typically ranging from a few seconds for ^1^H to minutes in the case of ^13^C or other heteronuclei) the increased signal can be detected using either one hard or a series of small angle radio-frequency (rf) pulses. Unfortunately, due to its irreversible nature, dDNP can only scratch the surface of the full spectroscopic potential of hyperpolarized liquid-state NMR with experiments that require numerous consecutive acquisitions for either phase cycling or multidimensional detection schemes. Multidimensional NMR, despite causing a revolution when it was introduced already 50 years ago, thus remains mostly incompatible with dDNP to this day in contrast to many other hyperpolarization strategies.

In the case of parahydrogen-induced hyperpolarization (PHIP), the introduction of the signal amplification by reversible exchange (SABRE) technique enabled continuous room-temperature hyperpolarization of specific targets by means of p-H_2_ acting as an inexhaustive source of spin order (*17*–*21*). Also, chemically induced DNP (CIDNP) (*22*–*24*) and optical pumping (OP) (*25*, *26*) can continuously hyperpolarize specific targets via a number of photoactive molecules and noble gases, respectively, in relatively easy-to-operate experimental conditions. In very recent studies, photo-CIDNP (*27*) and SABRE (*28*) were successfully adapted for in situ benchtop hyperpolarization, yielding high ^1^H and ^15^N signal enhancement, respectively, which can be integrated into standard NMR experiments including the use of signal averaging. Yet, despite their reversible character and less demanding hardware requirements, hyperpolarization via PHIP, SABRE, CIDNP, and OP remains highly target specific. The presence of specific chemical interactions between polarizing agents and the respective molecular targets is a key ingredient to activate the hyperpolarization mechanism. More recently, indirect methods were developed in which hyperpolarization initially produced by DNP (*29*) or PHIP at room temperature is transferred by nuclear Overhauser effect (NOE) after mixing the polarized source and unpolarized target molecules. While these methods have, to some extent, the ability to deliver nonselective hyperpolarization (with varying efficacy due to the NOE transfer), they remain destructive by mixing and diluting.

DNP-induced hyperpolarization at low temperature produces hyperpolarization that spontaneously propagates among homonuclear spins through spin diffusion (*30*), which thrives on energy-conservative dipolar nuclear flip-flops. This allows for the efficient redistribution of nuclear polarization homogeneously to any complex mixtures of targets in the sample independent of its chemical nature. Still, dDNP remains destructive by dilution, therefore single shot.

A major scientific challenge remains to consolidate the universality of DNP and the benefits of multidimensional NMR. The introduction of ultrafast two-dimensional (2D) sequences (*31*–*33*) to perform homonuclear 2D NMR acquisitions in a single scan after dissolution illustrates previous efforts to achieve this goal. However, ultrafast NMR requires strong gradient pulses that affect both spectral resolution and sensitivity, thus lowering the benefits of signal gain by hyperpolarization. On top, not all multidimensional experiments are amenable to ultrafast NMR to this day.

Inspired from previous work from Griffin’s (*34*) and Kentgens’ (*35*, *36*) groups, we are presently working on the development of widely accessible and recyclable DNP, with the additional aim of operating without dilution nor radical contamination, in an all-in-one compact system that will ultimately be installed on the bench and, for example, directly connected to a benchtop NMR system. This remote goal may ensure in 3 to 5 years compatibility of hyperpolarization with multi-scan analysis of liquid samples, aimed at advancing NMR in a chemical and metabolomic context.

We propose an instrumental setup designed to generate a pure and replenishable source of hyperpolarization, for a hyperpolarized flow (HypFlow) DNP methodology. This resembles continuous flow liquid-state Overhauser DNP (*37*, *38*), in which the protons of the solvent (and possibly other molecules with labile proton sites) can be enhanced by up to two orders of magnitude. Additionally, the flow liquid-state DNP approach could hyperpolarize other nuclei directly, such as ^19^F (*39*) or ^31^P (*40*), or indirectly such as ^13^C with PENDANT polarization transfer schemes (*41*).

**Fig. 1.**
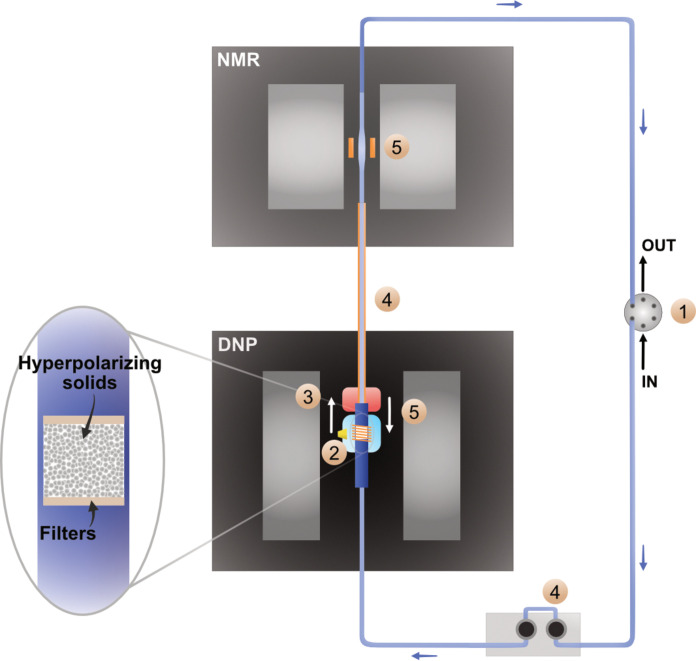
Scheme of the prospective HypFlow DNP system with its different parts. In (1), the ~600-μl sample loop is filled with the solution through port IN and OUT of a six-way port valve. When the loop is filled, the valve turns and closes the loop. A portion of the sample (in white) impregnated in the hyperpolarizing mesoporous material, retained between two filters (see zoom), is then frozen (2) at 77 K in the cryostat (in light blue) where DNP occurs upon frequency-modulated microwave irradiation (μwave horn in yellow) at 1 T. After the maximum level of polarization is reached, the DNP flow cell (in dark blue) is lifted (3) with a stepper motor (not represented here) to the sample melting system (in red) and the solution is rapidly melted. The high-pressure dosing pump is then activated (4) to move the liquid in the sample loop, thus transferring the hyperpolarized portion of the solution into the NMR coil. Here, the liquid-state analysis (5) occurs at 2 T, while the DNP flow cell is lifted down again to freeze another portion of the sample and perform simultaneous DNP.

In our approach, the DNP polarizer operates at readily accessible conditions of 1 T and 77 K and features ^1^H→^13^C cross polarization (CP). DNP at low temperatures, in the frozen state, enables a broader and more general distribution of gains of polarization via spin diffusion, which requires only vicinal dipolar contacts in the solid state. Moreover, the use of solid-state CP provides a straightforward extension to virtually any heteronuclear spins. Ultimately, spin diffusion and CP provide a more versatile process for the hyperpolarization of complex mixtures. Figure 1 schematically summarizes the envisioned HypFlow DNP approach aiming to replenish the hyperpolarization of target analytes flowing through a closed loop. The DNP polarizer uses liquid nitrogen (l-N_2_) instead of liquid helium as a coolant, and a permanent magnet instead of a superconducting magnet. This allows a compact design with dramatically reduced running costs. Similar to what was previously demonstrated in liquid-state or dDNP (*38*, *42*, *43*), HypFlow incorporates a heterogeneous stationary phase composed of porous materials (e.g., silica or polymer based) containing covalently immobilized radicals, in which the analytes of interest can be impregnated (*42*–*45*) to circumvent the dissolution and dilution of the sample and the use of radical and glassing agent contaminants. The analytes get impregnated in the mesoporous hyperpolarizing solids (HYPSOs) that fuel hyperpolarization through covalently attached radicals once the heterogeneous phase has been cooled to 77 K. The use of immobilized radicals blocked in a flow cell by means of micrometer-sized filters is critical in keeping the analyte solution radical free and in preserving hyperpolarization, as already demonstrated in the context of dissolution DNP (*43*). Ultimately, beyond this article’s scope, a rapid melting step is envisioned (*34*, *35*), after which pure analyte solutions will be extruded with a high-pressure liquid pump, similar to what has been achieved by Han and Münnemann and coworkers in liquid-state DNP (*37*, *38*). Our ultimate goal is to flow a pure hyperpolarized solution toward a benchtop or high-field NMR measurement, while most of its enhanced polarization is preserved.

The present study focuses on a first step toward our aim of developing a widely accessible and recyclable DNP alternative, namely, the development of the DNP polarizer and the initial quantification of solid-state DNP performances for both ^1^H and ^13^C at *B*_0_ = 1 T and *T* = 77 K with free radicals in solution as well as grafted onto HYPSOs. This is a crucial step to assess the overall performance of the approach, which is unpredictable as DNP experiments, preceding dissolution, are usually performed at much lower temperatures (1 to 4 K) and/or at much higher magnetic fields (3 to 7 T). The instrumentation presented here was built precisely to access this information and is suited for static DNP measurements. The DNP enhancements on ^1^H and ^13^C were first quantified with nitroxide radicals in a frozen solution. The feasibility of enhancing the sensitivity of impregnated solutions in porous matrices with covalently attached nitroxide radicals was also proven in those DNP conditions with an enhancement of ~62 for protons in water, and the high polarization subsequently transferred to ^13^C spins of [1-^13^C] sodium acetate, with an extended hyperpolarization lifetime compared to that of proton. This is a critical milestone that opens the way to our HypFlow approach.

## RESULTS

### Design of the benchtop DNP polarizer

**Fig. 2.**
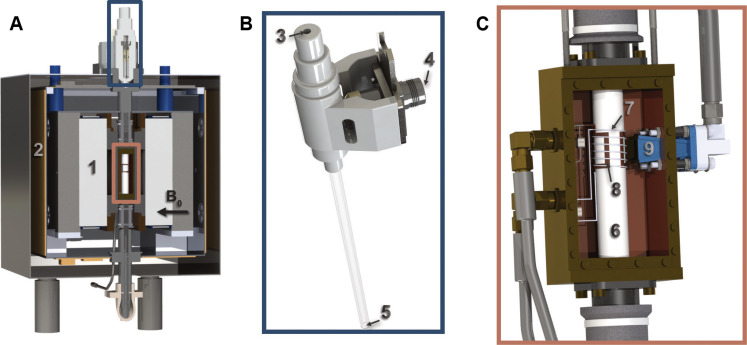
Overview of the design of the benchtop DNP polarizer. (**A**) Frontal cut of the DNP polarizer. The two plates of the 1-T permanent magnet (1) producing a horizontal magnetic field are attached by a stainless-steel frame. The magnet is temperature stabilized with the heating mats (2) and additional thermal insulation (not represented here). Two pairs of feet create a space underneath the polarizer for the nitrogen exhaust. (**B**) The cryostat is suited to fit an EPR tube inserted in the sample hole (3) and is cooled by the continuous flow of nitrogen from the input (4) to the output (5). (**C**) The probe cavity is fitting between the magnets structure and is fixed in the polarizer. The transverse cut of the DNP NMR probe shows the copper housing, the Teflon tube (6) on which the ^13^C solenoid rf coil (7) and the ^1^H saddle rf coil (8) are wounded. The rectangular horn (9) is placed in front and as close as possible to the NMR coils and directs the microwaves onto the sample location. Reproduced from (*60*) with permission from the PCCP Owner Societies.

The benchtop DNP polarizer, visualized in Fig. 2A and in fig. S1, was co-developed in collaboration with Bruker BioSpin serving as the prototype for the HypFlow system. The dimensions of the DNP polarizer are only 52 cm × 35 cm × 36 cm (*l* × *w* × *h*) encasing a temperature-stabilized 1-T permanent magnet. The static magnetic field (*B*_0_) is orientated horizontally and provides a 22-ppm (parts per million) homogeneity over the sample volume (200 μl) suited for performing both DNP and static solid-state NMR experiments. Inside the polarizer, a DNP insert includes (i) a cryostat for operating at a base temperature of 77 K, (ii) a microwave transmitting antenna to induce DNP transfer from electrons to nuclear spins, and (iii) ^1^H and ^13^C rf coils to perform both CP transfer and detect the respective hyperpolarized NMR signals. The samples under investigation can be analyzed in a conventional 4-mm electron paramagnetic resonance (EPR) tube, which can be accommodated inside the DNP cavity.

To freeze the sample, a vacuum-sealed glass insert (see Fig. 2B) is continuously supplied with nitrogen gas cooled and partly liquified by a heat exchanger. The base operating temperature of the cryostat reaches down to 77 K, which is substantially higher than for conventional dDNP experiments operating at liquid helium temperatures at around 1.2 to 1.6 K. While this compromises the final achievable nuclear spin polarization due to an electron polarization of *P*(e) ≈ 0.85% [instead of *P*(e) > 99% under dDNP conditions], it provides more magnetization per unit of time (buildup times on the order of a second instead of close to an hour) and overall a dramatically faster DNP turnover, thus enabling future implementation for replenishable hyperpolarization with freeze/melt cycles. The full operation and temperature monitoring inside the cryostat is described in Materials and Methods.

A custom-made Kα-band microwave source generating frequencies in the range of 26.6 to 28.8 GHz allows for polarization transfer from electrons to nuclear spins, with optional sinusoidal or triangular frequency modulation, and a maximal power of 5 ± 2 W depending on the frequency. Once generated by the microwave oscillator and amplified by a solid-state amplifier, the microwaves are transmitted to the DNP cavity by means of a coaxial transmission line. Next, a coaxial to WR-28 rectangular waveguide transition excites the TE_01_ rectangular microwave mode. A standard gain microwave horn finally acts as an antenna and directs the microwave to irradiate the frozen sample placed inside the benchtop polarizer.

The design of the complete DNP NMR probe is schematically visualized in Fig. 2C and consists of a double coil configuration with a saddle coil made of copper tape for proton detection (ωH1/2π = 42.7 MHz) and a solenoid coil made of 0.5-mm copper wire for carbon detection (ωC13/2π = 10.7 MHz). Both rf coils were wound around a Teflon tube, chosen for (i) avoiding electrical contact between the two coils, (ii) allowing for efficient rf and microwave penetration, and (iii) avoiding a ^1^H background signal to guarantee accurate DNP enhancement calculations. Two orthogonal magnetic fields (*B*_1_) perpendicular to the static field (*B*_0_) are produced by the saddle coil (2.9 kHz/W^½^ for proton) and the solenoid coil (3.9 kHz/W^½^ for carbon). The *B*_1_ fields are geometrically decoupled using perpendicular coils, which avoid crosstalk between the two channels. With this configuration, double rf irradiation at relatively high power (typically 10 to 100 W) and through prolonged periods of contact (typically 1 to 10 ms) is feasible, which is required to perform efficient polarization transfer by ^1^H→^13^C CP technique. Further benchmarking of the probe performances is described in Materials and Methods.

### ^1^H DNP performances on a frozen DNP solution

The DNP performances of the compact 1-T benchtop polarizer operating at 77 K were first evaluated using a 200-μl solution of 50 mM 4-hydroxy-2,2,6,6-tetramethylpiperidine-1-oxyl (TEMPOL) in 2:2:6 H_2_O:D_2_O:DMSO-d_6_ (hexadeuterodimethyl sulfoxide) (v:v:v). This chemical formulation is acknowledged for its good glassing capabilities (*46*, *47*), reproducibility, and inexpensiveness compared to formulation with deuterated glycerol. Since previous work has assessed the effectiveness of such sample formulation under conventional DNP conditions (*48*, *49*), it was therefore used to ensure comparability on the first hyperpolarization results.

**Fig. 3.**
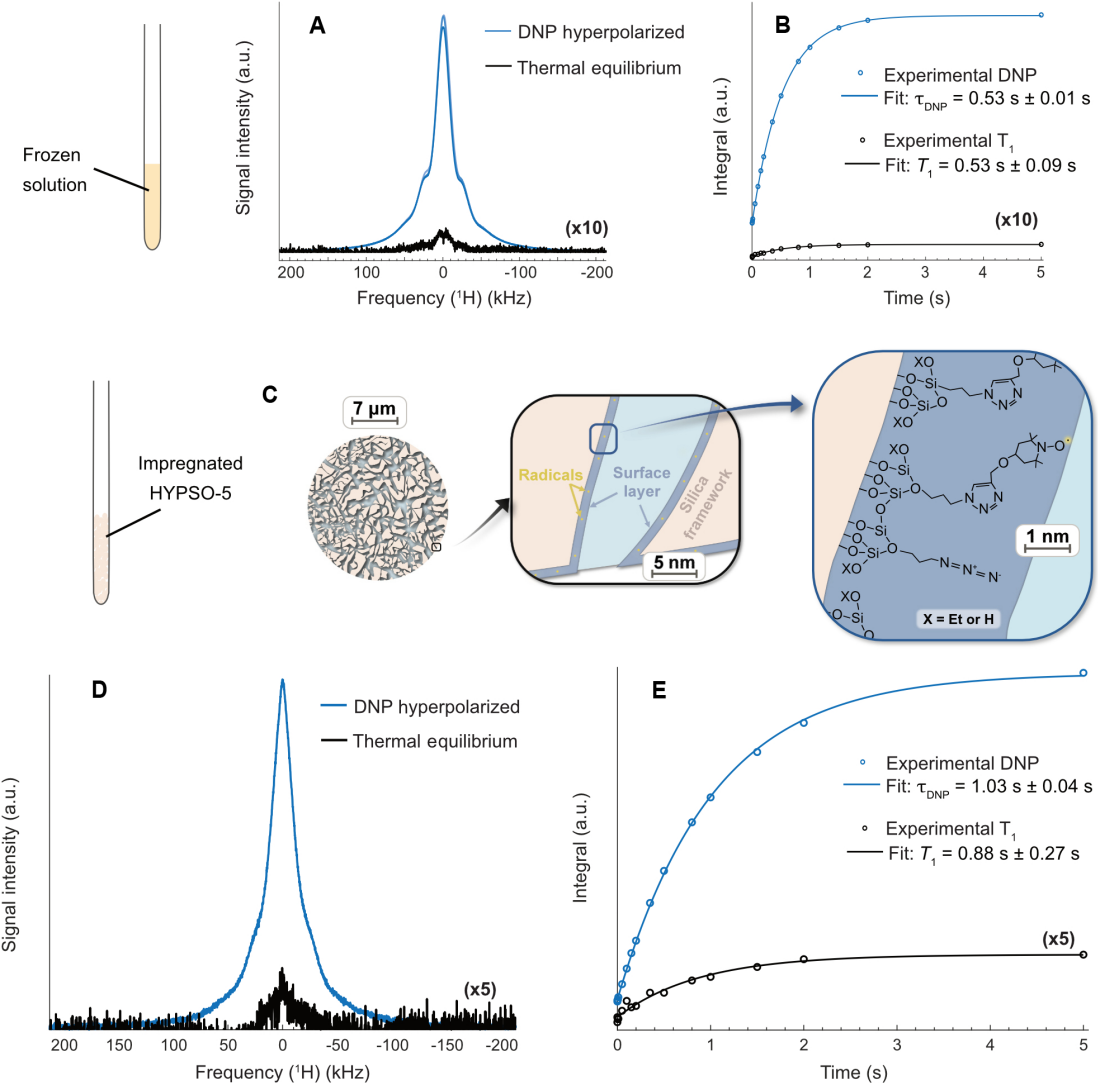
Overview of the ^1^H hyperpolarization performance in frozen DNP solution and hyperpolarizing matrices. (**A**) ^1^H DNP experiments performed with the benchtop polarizer at 77 K and 1 T for a 2:2:6 H_2_O:D_2_O:DMSO-d_6_ (v:v:v) DNP solution with 50 mM TEMPOL. The hyperpolarized signal (in blue) corresponds to the maximal level of polarization acquired while applying microwaves at a frequency *f*_μw_ = 28.16 GHz with a triangular frequency modulation of Δ*f* = ±20 MHz at a rate *f*_mod_ = 60 kHz and a power *P*_μw_ = 5 W. The DNP experiment was performed three times with three different batches of the solution. The enhanced signal is compared with the TE signal of proton in the same sample shown in black without microwaves. (**B**) ^1^H polarization buildup curve (in blue) and *T*_1_ relaxation curve (in black). Signals were fast Fourier transformed and integrated, and the time courses of the integrals were fitted with mono-exponential functions. (**C**) ^1^H DNP experiments for a 2:8 H_2_O:D_2_O (v:v) solution impregnated in HYPSO-5 with a radical loading of 43 μmol cm^−3^. Schematic view of a mesoporous HYPSO-5 silica bead with a zoom on the pore composition and the sample composition of the pore layer in contact with the solution and containing the radicals. More information on HYPSO-5 can be found in fig. S2. (**D**) The hyperpolarized signal (in blue) corresponds to the maximal level of polarization acquired while applying microwaves at a frequency *f*_μw_ = 28.17 GHz with a triangular frequency modulation of Δ*f* = ±20 MHz at a rate *f*_mod_ = 60 kHz and a power *P*_μw_ = 5 W. The enhanced signal is compared with the TE signal of proton shown in black without microwaves. (**E**) ^1^H polarization buildup curve (in blue) and *T*_1_ relaxation curve (in black).

Figure 3A shows the thermal equilibrium (TE) signal of the frozen DNP solution (in black) acquired without microwave irradiation and the DNP-enhanced signal acquired on three different samples (three lines in blue hardly discernible) and measured 5 s after switching on the microwaves. After optimizing the microwave frequency as described in Materials and Methods, DNP yielded a solid-state signal enhancement of ε = 100 ± 6 if compared to the fully relaxed TE signal of the 22 M water protons. This value corresponds to a proton spin polarization of *P*(^1^H) = 0.13%, while the Boltzmann distribution of the electron in those conditions fixes a theoretical maximum achievable polarization of *P*(e) = 0.84%. The first attempt of enhancing the signal within our prototype polarizer offers a satisfying polarization transfer with a signal boosted by two orders of magnitude.

In Fig. 3B, the DNP buildup time (in blue) and the longitudinal relaxation time (in black) were acquired with a saturation recovery experiment with and without microwave irradiation, respectively. The proton polarization under DNP builds up with a time constant of $\tau_{\text{DNP}}$ = 0.5 s, similar to the TE longitudinal relaxation time *T*_1_. This can be compared with what was reported at higher fields of 3.38 T and *T* ~ 10 K in static DNP (*50*), where an enhancement of 60 using 40 mM TEMPOL in a nondegassed sample was achieved through cross effect and spin diffusion with $\tau_{\text{DNP}}$ ~ 20-s buildup time constants. The sensitivity attained here in 5 s would be attainable without DNP by scan accumulation over 13 hours.

### ^1^H DNP performances using hyperpolarizing matrices

Radical immobilization on mesoporous matrices is an essential element of our approach to generating contaminant-free and pure hyperpolarized analytes. HYPSO-5 matrices (*43*) consist of mesoporous (pore diameter of about *d* = 4 to 5 nm) silica beads of 15 μm diameter, coated with a silica layer containing TEMPO radicals (43 μmol cm^−3^) for this study. The beads contain multiple interconnected pores, as shown in Fig. 3C, that allow for fast penetration of the solution in and out of the material. The HYPSO-5 beads exhibit a silica coating layer, allowing a homogeneous distribution of immobilized radicals across the active surface of the silica-based material, including the mesopores. The immobilized radicals residing on the pore surfaces mimic the random distribution of radicals of the frozen DNP solution that is proven to be optimal (*43*). In the frozen state, polarization transfer happens through direct DNP to the nuclear spins nearby the immobilized radicals, followed by ^1^H spin diffusion to the bulk. Assuming a ^1^H spin diffusion constant *D* = 54 nm^2^ s^−1^ (see the details in section S6) (*51*), ^1^H polarization would typically spread during the 5 s of the DNP buildup measurement over lengths on the order of *L* = √4*Dt* = 32 nm. This is larger than the pore diameter; therefore, spin diffusion is believed to efficiently spread the acquired proton polarization throughout the entire pores at a faster pace than the DNP buildup.

The HYPSO-5 DNP performances were tested by impregnating 73.7 mg of powder with the exact pore volume of *V*_p_ = 46.4 μl of a 2:8 H_2_O:D_2_O (v:v) solution and by freezing it inside the cryostat operating at 77 K placed inside the 1-T DNP polarizer. In Fig. 3D, the TE signal (black line) is depicted with the DNP-enhanced signal (blue line) measured 5 s after switching on the microwaves at *f*_μw_ = 28.17 GHz with a triangular frequency modulation of Δ*f* = ±20 MHz at a rate *f*_mod_ = 60 kHz and a power *P*_μw_ = 5 W. The solid-state signal enhancement of 62 exhibits favorable first results for hyperpolarization using the mesoporous solids in combination with the benchtop DNP polarizer. The sensitivity attained here in 5 s would be attainable without DNP by scan accumulation over 5 hours.

In Fig. 3E, the hyperpolarization buildup (in blue) was acquired with a saturation recovery experiment under microwave irradiation and shows a polarization buildup time of $\tau_{\text{DNP}}$ = 1.03 s ± 0.04 s, i.e., twice that with TEMPOL in a homogeneous solution. The nuclear spin-lattice relaxation of the protons residing in the mesopores of HYPSO-5 (in black) has a characteristic decay time of *T*_1_ = 0.88 s ± 0.27 s, about 1.65 times that observed in the homogeneous case. The reduced DNP efficiency using HYPSO-5 might come from several factors.

1)The radical loading in the HYPSO material is somewhat less (443 ^1^H spins to 1 electron) than in the noted free solution (516:1) and may not be optimal. Further concentration studies are required to assess the ultimate potential of DNP efficiency with these advantageous porous materials.

2)The distribution of the radicals may not be as homogeneous, compared to the statistical distribution of radicals in a flash-frozen DNP solution. Such distribution inhomogeneities may potentially be unraveled in the future by distance measurements through pulsed EPR measurements.

3)The presence of larger pores (larger than 5 nm as measured by N_2_ adsorption; see fig. S2) may exacerbate the limiting aspect of spin diffusion. Spin diffusion may thus act as a bottleneck, limiting the spread of polarization from radicals on the material walls, out and across solution trapped in oversized pores.

4)Impregnation of HYPSO-5, although performed for the exact pore volume, might be suboptimal, with a fraction of the solution residing outside of the pores, in the interstices between HYPSO-5 particles, where no radicals are present, and thus where DNP is inactive.

To further optimize DNP performance and explore the limiting role of spin diffusion, we will perform further experiments with a range of radical loadings and pore sizes in HYPSO-5 in a future study.

### ^13^C DNP performances in hyperpolarizing matrices and frozen solution

The ability of the benchtop DNP polarizer to efficiently hyperpolarize ^13^C spins is a crucial goal. This follows on the ^1^H polarization studies above, as DNP of protons may be used as a source for CP-based transfer to ^13^C (or other nuclei). Carbon is a ubiquitous element in countless molecules and has the advantage that its chemical shift does not overlap as much as proton signals, and it has a slower *T*_1_ relaxation. Yet, due to the low natural abundance of its magnetically active ^13^C isotope, it remains challenging to measure, especially using traditional benchtop NMR systems. Hyperpolarization, therefore, can render ^13^C an attractive target for NMR applications from low to high field, notably in reaction monitoring (*52*, *53*) and metabolomic studies (*54*).

**Fig. 4.**
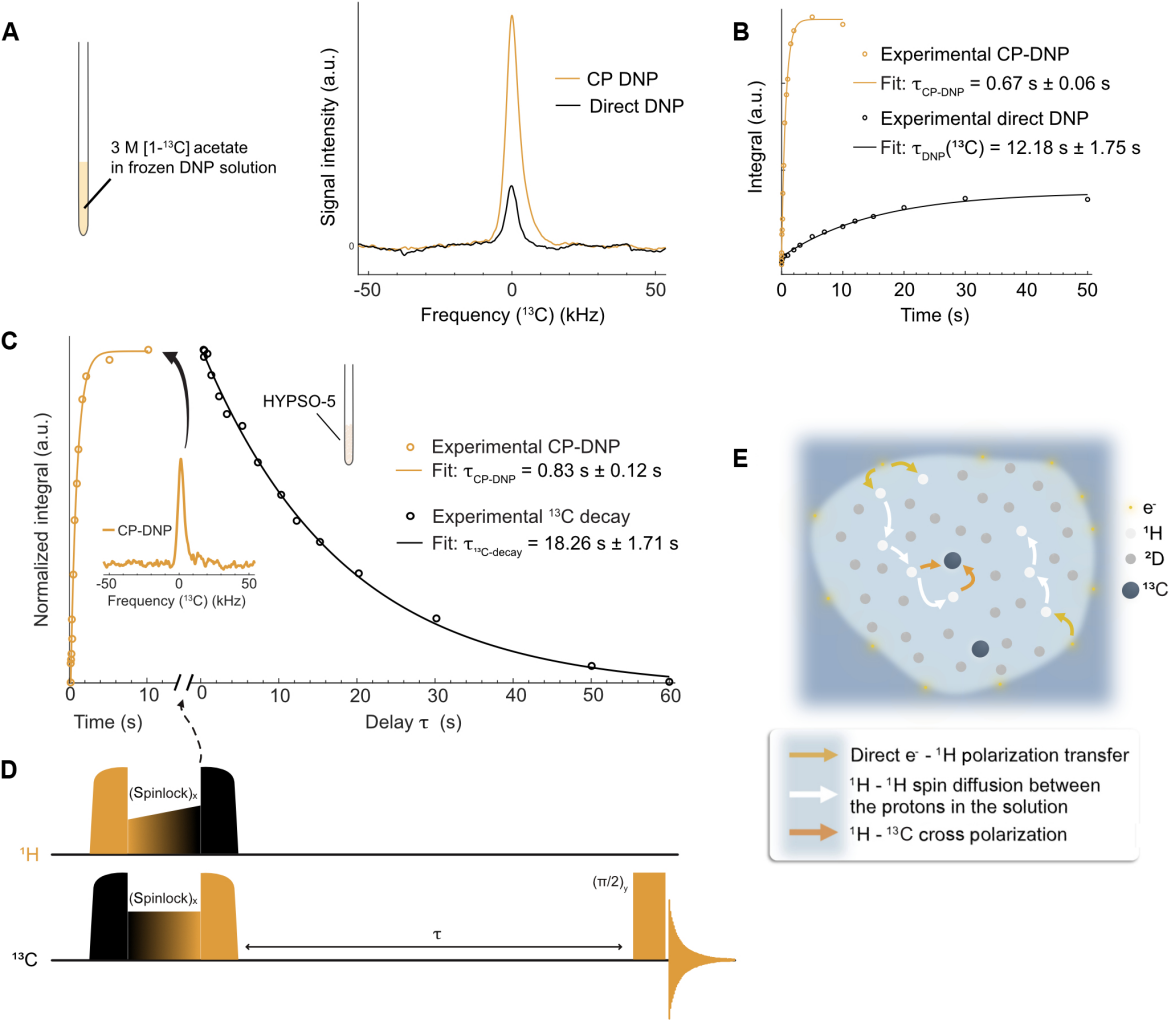
Overview of the CP-DNP performances in both frozen DNP solution and impregnated solution in HYPSO-5. (**A**) ^13^C CP-DNP (yellow) and direct ^13^C DNP (black) experiments at 77 K and 1 T of 3 M [1-^13^C] sodium acetate in 2:2:6 H_2_O:D_2_O:DMSO-d_6_ (v:v:v) with 50 mM TEMPOL measured at *f*_μw_ = 28.15 GHz with a triangular frequency modulation of amplitude Δ*f* = ±20 MHz at a rate *f*_mod_ = 60 kHz and a power *P*_μw_ = 5 W. (**B**) Respective polarization buildup time. (**C**) ^13^C CP-DNP experiment at 77 K and 1 T of 3 M [1-^13^C] sodium acetate in 2:8 H_2_O:D_2_O (v:v) impregnated in HYPSO-5 with a radical loading of 43 μmol cm^−3^ measured at *f*_μw_ = 28.35 GHz with a triangular frequency modulation of amplitude Δ*f* = ±20 MHz at a rate *f*_mod_ = 60 kHz and a power *P*_μw_ = 5 W. The buildup time (yellow) was measured with an increasing CP-DNP buildup time as shown in the pulse sequence in (**D**). The adiabatic half-passage chirp pulses with half WURST (wideband, uniform rate, smooth truncation) sweep over 100 kHz in 250 μs with 500 points in both channels (50 W for ^13^C and ^1^H). Contact is realized with 1.5-ms contact pulses with a square pulse on ^13^C (26.4 kHz at 80 W) and a ramp 80% to 100% on ^1^H (24.8 kHz→27.8 kHz at 50 W) to compensate for *B*_1_ field inhomogeneities. An additional adiabatic pulse puts the magnetization on the *z* axis, and the delay τ is incremented before each acquisition to follow the ^13^C decay (black). (**E**) Scheme of the impregnated HYPSO-5 pore where the radicals are fixed.

We assessed the ^13^C DNP performances of the benchtop polarizer first using a standard sample of 3 M [1-^13^C] sodium acetate in a 200-μl solution of 50 mM TEMPOL in 2:2:6 H_2_O:D_2_O:DMSO-d_6_ (v:v:v). In Fig. 4A, both direct ^13^C DNP and ^13^C CP-DNP were tested and compared. The first method consists of the direct transfer of the electron polarization to the ^13^C spins by means of microwave irradiation, while the second uses ^1^H spins as an intermediate polarization carrier. Here, electron polarization is first transferred to nearby ^1^H spins by microwave irradiation and then to ^13^C by CP sequences (*55*, *56*). The matching conditions for optimal CP polarization transfer are detailed in Materials and Methods.

The buildup time for ^13^C direct DNP was measured with a saturation recovery experiment with DNP and was found to be $\tau_{\text{DNP}}$(^13^C) = 12.18 s ± 1.75 s as shown in Fig. 4B. The CP-DNP buildup time for ^13^C was measured with the same method and exhibited an 18-fold faster buildup time, with $\tau_{\text{CP}-\text{DNP}}$ = 670 ms ± 60 ms, a value primarily determined by the ^1^H DNP buildup time. In addition, CP-DNP also resulted in a four times larger DNP enhancement compared to direct DNP. A nitroxide radical such as TEMPOL was chosen with CP in mind, as these radicals exhibit EPR resonances broader than the ^1^H NMR frequency, and thus well-suited to hyperpolarize ^1^H nuclei and enable CP as the best approach for relayed hyperpolarization of heteronuclei like ^13^C (*46*). Neither the ^13^C signal enhancement nor *T*_1_(^13^C) could be directly estimated because of a lack of ^13^C sensitivity at TE signals.

For the long-term goals of rapid cycled freeze/melt DNP and NMR observation, it is also important to assess the quality of ^13^C polarization achievable by the CP-DNP method using HYPSO-5 as the polarizing agent. The same HYPSO-5 as in the previous section was used, here with 66.6 mg of powder impregnated with 42.0 μl of a solution containing 3 M [1-^13^C] sodium acetate in 2:8 H_2_O:D_2_O (v:v). Direct ^13^C DNP with HYPSO-5 did not yield observable signal even when averaging 896 scans over 15 hours. However, with ^1^H→^13^C CP-DNP and HYPSO-5 (see Fig. 4C), one could obtain a ^13^C spectrum with a signal-to-noise ratio of ~30 over only 40 s with eight scans. The dependence on buildup time for the CP-DNP is also shown (in yellow), measured using a saturation recovery experiment with DNP as represented in Fig. 4D. The resulting $\tau_{\text{CP}-\text{DNP}}$ = 0.83 s ± 0.12 s is similar to the proton buildup time and, again, slightly slower than in the homogeneous solution. When HYPSO-5 was impregnated with the same 2:8 H_2_O:D_2_O (v:v) solution but without [1-^13^C] sodium acetate, the buildup time further slowed down. This observation is probably due to the presence of additional protons that accelerate the spin diffusion process. Finally, we measured the ^13^C relaxation after CP (black curve in Fig. 4C) by using an increasing delay after the CP transfer. To our surprise, the signal did not decay to a detectable enhanced direct DNP equilibrium as it is the case in conventional DNP solutions. Instead, the signal decayed to a very small value below the detection limit, with a time constant of T1(C13) = 18.26 s ± 1.71 s, which is 1.5 times longer than the nuclear spin-lattice relaxation time in the homogeneous solution.

This extension in ^13^C relaxation times is a critical key advantage of our approach using HYPSO polarizing matrices over DNP solutions, i.e., such that matrices can ensure very slow loss of hyperpolarization even after a sample is melted and sent on for solution-state observation. There are several underlying reasons for the advantage. First, ^13^C spin diffusion is considerably slower than ^1^H spin diffusion. The diffusion coefficient is proportional to the nuclear spin concentration and follows the equation *D* ∝ γ^4^, where γ is the gyromagnetic ratio. Therefore, one can expect in our conditions *D*(^13^C) ∼ 0.02 nm^2^ s^−1^ (considering a 0.1 ^13^C-to-^1^H concentration ratio), which leads to a diffusion length in 5 s of only 0.3 nm, insufficient to efficiently relay paramagnetic relaxation from the surface of the pores. Second, radicals are predominantly situated inside the layer located at the surface of the pores (see Fig. 4E), where the ^13^C concentration is only present in the 1.1% natural abundance. This very small quantity of ^13^C nuclear spins proximal to the electrons results in inefficient paramagnetic relaxation or DNP transfer from the surface layer to the frozen solution. The CP-DNP offers the advantage of a fast ^13^C hyperpolarization, even at long distances from the polarizing agents in the pores of the polarizing matrices. Thus, one can both achieve fast, efficient hyperpolarization of ^13^C and, because of the relative separation of electron and nuclear spins, eventually preserve and use the hyperpolarization at low loss upon conversion to the solution state. These are often oppositional goals, but here brought into harmony with sample format (using HYPSO material) and method (CP) that allow quick buildup of hyperpolarization that is robust to melting and then transfer for solution-state NMR observation. It is worth noting that the measured rates were observed on nondegassed samples. Subsequent experiments involving oxygen removal may help further extend relaxation times and better preserve hyperpolarization.

## DISCUSSION

Here, we have successfully demonstrated the feasibility of ^1^H DNP and ^13^C CP-DNP in glassy DNP solutions and with HYPSO-5 polarizing matrices, using a tabletop DNP polarizer operating at *B*_0_ = 1 T and *T* = 77 K. Our initial results show a substantial boost in signal for both proton and carbon nuclear spins, achieving two-order-of-magnitude enhancement on ^1^H in less than a second, corresponding to four-order-of-magnitude time gain in signal averaging. The use of tailored mono- or bi-radicals possibly with longer spin-lattice relaxation times may lead to even greater performances in analogy to magic angle spinning DNP experiments (*57*). We next plan to implement fast melting with various strategies as successfully reported by Griffin’s and Kentgens’ groups (*34*, *35*). Additionally, the use of HYPSO-5 is essential as it offers two important features: (i) a high-speed direct ^1^H and ^1^H→^13^C CP assisted DNP and, most importantly, (ii) spin diffusion–limited slow ^13^C relaxation, which may limit hyperpolarization losses upon melting and transfer. While HYPSO-5 (*d* = 5 nm) can typically fit up to 50-kDa molecules (*58*), we are currently investigating the use of HYPSOs with larger pores as well as hyperpolarizing polymers (HYPOPs) (*45*) with pores in the micrometer range. In our setting, liquid-state NMR will be ultimately performed in an adjacent benchtop NMR spectrometer operating at *B*_0_ = 1.88 T and room temperature, in which enhancements of two orders of magnitude are foreseen, corresponding to a time saving of typically four orders of magnitude, if considering a similar repetition time of 5 s for liquid-state NMR and DNP-enhanced NMR (including freeze, DNP, melt, and flow). This work paves the way for our final goal of integrating this DNP strategy with a system enabling on-the-bench multi-scan liquid-state NMR acquisitions on hyperpolarized mixtures of small molecules. Further improvements are foreseen, for example, using higher microwave power or tailored mono- or bi-radicals. The use of more elaborate cooling systems such as helium compressors could also be considered in the future to further cool down the sample and achieve higher nuclear spin polarizations.

## MATERIALS AND METHODS

### ^1^H and ^13^C DNP sample preparation with free TEMPOL radicals

The free radicals TEMPOL (99.96% purity, CAS 2226-69-2), D_2_O (99.5% deuteration, 99.96% purity, CAS 7789-20-0), and the glassing agent DMSO-d_6_ (99.5% deuteration, 99% purity, CAS 2206-27-1) were purchased from Sigma-Aldrich. The DNP solution used for testing the ^1^H hyperpolarization performance of the benchtop polarizer was prepared by weighing 4.3 mg of TEMPOL and dissolving the radicals in a glass-forming mixture of 300 μl of DMSO-d_6_ and 100 μl of D_2_O with 100 μl of H_2_O as the analyte. This resulted in a concentration of 50 mM TEMPOL in the DNP solution comprising a final volumetric ratio of 2:2:6 H_2_O:D_2_O:DMSO-d_6_ (v:v:v). An aliquot of 210 μl of the DNP solution was pipetted and introduced into the EPR tube (Wilmad quartz EPR tube outside diameter 4 mm, L 250 mm). The sample was then shock frozen by rapid introduction of the EPR tube in the DNP cryostat precooled to 77 K. The DNP solution used for testing the ^13^C hyperpolarization performance was prepared starting from weighing 198 mg of [1-^13^C] sodium acetate (CAS 23424-28-4) with a ^13^C isotope purity of 99% obtained from Eurisotop. It was dissolved in a mixture of 480 μl of DMSO-d_6_, 140 μl of D_2_O, and 160 μl of H_2_O. TEMPOL (6.9 mg) was weighed separately and dissolved in 20 μl of D_2_O. After sonication during 10 min at 30°C and vortex mixing, complete dissolution of the [1-^13^C] sodium acetate in the glass-forming DNP solution, the solution of TEMPOL was added and mixed with vortex mixing.

### ^1^H and ^13^C DNP sample preparation with HYPSO-5

The HYPSO-5 powder used in this work was synthesized following the procedure described in (*43*). The resulting HYPSO was derived from commercially available mesoporous silica beads (SiliaSphere) coated with a uniform silica layer containing TEMPO radicals (43 μmol cm^−3^). The concentration of radicals contained in HYPSO-5 was quantified by recording X-band continuous wave EPR spectra at room temperature. The average pore diameter and pore volume were measured to be 4 to 5 nm and 0.63 cm^3^ g^−1^, respectively, and were determined via N_2_ adsorption-desorption (performed at 77 K using BELSORP-max after degassing the sample at 408 K under 10^−5^ mbar vacuum for 15 hours). For testing the DNP performance of HYPSO-5, a partially protonated 2:8 H_2_O:D_2_O (v:v) solution was impregnated in the pores amounting the total pore volume of the HYPSO-5 sample. In practice, the volume of HYPSO-5 required for performing DNP was calibrated to fill the entire NMR coil space analogs to 200 μl of DNP solution, without packing. This represented 73.7 mg of powder that was impregnated with 46.4 μl of the partially protonated solution to only fill the pores. For the impregnation, the solution was dropped onto the powder sitting in a watch glass, and mixed with the powder with a glass tip until it appeared dry again (capillary impregnation). The impregnated HYPSO-5 was then simply introduced into the EPR tube without packing, immediately after impregnation as no additional delay is needed to improve results. Finally, for ^13^C DNP inside HYPSO-5, 24.9 mg of [1-^13^C] sodium acetate (CAS 23424-28-4) with a ^13^C isotope purity of 99% obtained from Eurisotop was dissolved in 100 μl of partially protonated 2:8 H_2_O:D_2_O (v:v) solution. Again, complete dissolution of the [1-^13^C] sodium acetate was ensured by sonication and vortex mixing during 10 min at 30°C. As previously described here, 42 μl of the solution was used to impregnate and fill the pores of 66.6 mg of HYPSO-5 powder.

### ^1^H DNP experiments

The TE and DNP experiments were acquired with eight scans using hard π/2 rf pulses. TE signals of proton were acquired without microwave irradiation allowing proper polarization quantification. The carbon background from the Teflon was neglected as no signal could be detected by repeating the same measurement without sample. The microwave parameters were optimized for each sample as it depended on the temperature of the magnet at the time of the experiment, which, in turns, influence the value of static magnetic field *B*_0_. The background signal was first subtracted in Topspin from the TE experiment. The TE spectrum and the DNP spectrum were phased on zero-order phase. The first-order phase correction was systematically set to 0. The baseline offset was corrected manually. Both spectra were manually integrated. The enhancement corresponds to the ratio between the absolute integral of the enhanced signal and the background-subtracted TE signal

$$\varepsilon_{\text{DNP}}=\frac{I_{\text{DNP}}}{I_{\text{TE}}}$$ (1)

where both intensities are normalized by the number of scans and receiver gain. The buildup curves and spin-lattice relaxation experimental data were fitted in Matlab with a mono-exponential equation

$$I_{t}=I_{\infty }\left[1-\text{exp}\left(-\frac{t}{\tau_{\text{DNP}}\;\text{or}\;T_{1}}\right)+d\right]$$ (2)

where $\tau_{\text{DNP}}$ is the characteristic DNP buildup time and *T*_1_ is the longitudinal relaxation time constant. The error margins in Fig. 3 correspond to 95% certainty interval. The absolute polarization was computed by multiplying the Boltzmann polarization at *B*_0_ = 1 T and *T* = 77 K by the enhancement factor. To perform DNP at the optimal microwave frequency and optimal modulation parameters, the signal was empirically optimized by observing the signal through the Topspin multi-scanning “gs” interface.

### ^13^C DNP experiments

The hyperpolarized spectra were acquired both upon direct microwave irradiation and with CP. For direct ^13^C DNP, eight scans were used with hard π/2 rf pulses, resulting in an 8-min acquisition. For ^13^C CP-DNP, the rf pulse sequence in Fig. 4D (only the two first pulses) was used with eight scans, resulting in a 40-s analysis. The matching conditions for the CP polarization transfer were optimized with a spinlock power of 50 W on the ^1^H channel and 80 W on the ^13^C channel, yielding *B*_1_ fields of 27.8 and 26.4 kHz, respectively. The CP contact time was optimized to $\tau_{\text{CP}}$ = 1.5 ms, an optimal length limited by the proton nuclear spin relaxation in the rotating frame $T_{1\rho }$(^1^H). We measured $T_{1\rho }$(^1^H) = 1.2 ms ± 0.2 ms (see fig. S7). On the contrary, $T_{1\rho }$(^13^C) = 72 ms ± 50 ms is sufficiently long not to affect the CP efficiency. The ^13^C CP-DNP buildup rate was measured using a saturation recovery experiment upon microwave irradiation with eight scans and fitted with a mono-exponential fit in Matlab similar to the proton in Eq. 2. The ^13^C buildup rate, proportional to the relaxation rate, was measured with a saturation recovery experiment in DNP juice upon microwave irradiation with eight scans in 20 min. It was compared to the ^13^C relaxation rate measured using CP varying the delay between the end of the CP transfer and the acquisition acquired with eight scans in 25 min. This experiment was used to probe the ^13^C relaxation in the pores of HYPSO as sensitivity was not enough to measure it with a conventional saturation recovery experiment. For both of these experiments, the same fit in Eq. 2 was used with a positive or negative exponential decay.

### Polarizer magnet

Two neodymium magnets held by a stainless-steel frame and separated by a distance of 30 mm provide a horizontal permanent magnetic field of approximately 1 T with a homogeneity of 22 ppm over the sample volume (200 μl, 15 mm length). The magnetic stray field outside the housing falls below 0.5 mT, which makes the device safe to operate in most laboratory settings. Inside the housing, six independent heating elements and temperature sensors are connected to an external heating control unit to stabilize the temperature and therefore the *B*_0_ field. The field produced by the NdFeB magnets vary at −1200 ppm/°C. A mean temperature of 27.999 ± 0.006°C was used as a field setpoint, corresponding to a specific magnetic field of 1.004 T (±7.2 ppm). Overall, the system exhibited ^1^H linewidth of 924-Hz full width at half maximum in the liquid state. Cooling down the cryostat decreases the temperature inside the magnet, with an initial rate of about −0.7°C per hour (see fig. S3). This drift can be compensated by periodic adjustment of the NMR carrier frequency. In addition, after about 6 hours, the magnet temperature stabilizes to ±0.1°C (±120 ppm). Even this “settled” variation can still be a source of variation to CP-DNP performance, but fortunately, their slow timescale (tens of minutes) also allows compensation via adjusted NMR carrier frequencies. Further improvement in the future cryostat design will be needed to enable operating at fixed NMR and optimal microwave frequencies.

### Cryostat

The first version of the cryostat is based on a low-temperature vacuum-sealed glass insert operating at a base temperature of 77.1 K ± 0.4 K (measured at the sample position) by cooling with a flow of 40 slpm (standard liter per minute) of gaseous nitrogen (g-N_2_) at 0.5 bars passing through a l-N_2_ heat exchanger. The cryostat fits into a warm g-N_2_ insulating sleeve running through the magnet bore from top to bottom of the magnet enclosure and split into parts above and below the enclosure of the NMR probe (see Fig. 2) in such a way that the warm g-N_2_ also passes through the probe body. The rf coils and the microwave horn remain at ambient temperature. The cryostat accommodates a conventional 4-mm EPR tubes, held by a stopper element that seals nitrogen escape. It is supplied with the cooled gaseous nitrogen that enters the heat exchanger and that is then directed through a custom-made transfer line into the top of the cryostat. The cooled g-N_2_ passes downward along the length of the sample tube and is directed to the outside through a pipe at the bottom of the setup. The heat exchanger is inserted in a l-N2 Dewar, and the overall l-N2 consumption amounts to 100 liters per day when running continuous experiments. This improved to 15 liters per day over time. The constant flow of nitrogen in the cryostat ensures fast freezing of the sample when the tube is inserted in the bore of the glass tube of the cryostat. The temperature is monitored with a PT1000 temperature sensor placed close to the bottom of the EPR tube next to the sample and is acquired by an Arduino card and displayed with a dedicated Matlab application. The initial cool down starting from the housing temperature of 28°C takes approximately 5 min.

### Microwaves

The microwave frequency, the modulation waveform, and the modulation frequency are controlled with a graphical user interface (National Instruments). The microwaves are generated by a voltage-controlled oscillator (100 mW) and amplified afterward by a solid-state Qorvo amplifier chip up to a maximal power of 5 W ± 2 W, depending on the frequency. The microwave frequency range can be adjusted from 26.6 to 28.8 GHz with a resolution of 10 MHz. The waveform of the modulation is selectable between a triangular and sinusoidal mode for which the modulation amplitude and frequency can be adjusted from 1 to 200 MHz and 1 to 100 kHz, respectively, with a resolution of 1 kHz. Microwaves are transmitted to the DNP cavity by means of a coaxial transmission line (Mini-Circuits KBL-2FT-LOW+) with a 2.92-mm connector and a minimal insertion loss of around 1.29 dB. A WR-28 coax-to-waveguide transition from Pasternack then results in microwave transmission in TE_01_ mode (fig. S4). The maximal output power and modulation amplitude vary as a function of the frequency, yet correct amplification values are obtained from power calibration data (see table S1). At the sample position, the final magnetic field (*B*_1e_) strength of the microwave was estimated to an average value of 7.9 μT/W^½^ or 0.22 MHz/W^½^ over the sample volume by finite element numerical simulations in fig. S4, corresponding to *B*_1e_ ≈ 0.5 MHz when using the maximal output power of 5 W at the optimal microwave frequency for DNP. However, this field seems still insufficient, as noticed in fig. S5, where the integrated DNP signal versus microwave power is plotted for the 50 mM TEMPOL frozen solution and where no plateau is reached, which opens the opportunity to increase DNP efficiency even further in the future by merely increasing the microwave power or by improving the microwave coupling to the sample.

### DNP NMR probe

The ^1^H double saddle rf coil has a dimension of 20 mm height, 13 mm diameter, 3 mm of flat wire width, and an aperture angle of 120°, leading to an inductance close to 130 nH. Two fixed capacitors (ATC Ceramics 100A) of 47 and 58 pF soldered in parallel to the coil, lead to a total capacitance of 105 pF, and are used to tune the coil to roughly 42.7 MHz. The ^13^C rf coil has an overall dimension of 21 mm height and 14 mm diameter, leading to an inductance close to 350 nH. The carbon solenoid coil is made of a 0.5-mm copper wire wound in seven loops separated by 3 mm to let the microwave penetrate the coil and reach the sample. Two capacitors (ATC Ceramics 100A) of 330 and 47 pF soldered in parallel to the coil, lead to a total capacitance of 377 pF, and are used to tune the coil to roughly 10.7 MHz. Tuning and matching boxes [details published elsewhere (*59*)] are used to fine-tune the rf coils. They comprise two variable capacitors, one in parallel and one in series (Voltronics NMAT40HVE) attached after a 28-cm-long SMA cable connected to the ^1^H coil and 50 cm for the ^13^C coil.

Figure S6 (A and B) shows the reflectance in blue and transmittance in orange for both ^1^H and ^13^C channels. At the target frequencies, upon careful tuning and matching, ^1^H→^13^C and ^13^C→^1^H transmittances are respectively equal to −13 dB and −38 dB, and ^1^H and ^13^C reflectances are respectively equal to −61 dB and −36 dB, which was overall considered sufficient for our purpose. The quality factors of the ^1^H and ^13^C resonances (at −7 dB notch) are 88 and 67, respectively. The experimental nutation curves at room temperature for ^1^H and ^13^C are shown in fig. S6, C and D, respectively. The integral of the signal is plotted against the pulse duration for a fixed pulse power set to 50 and 200 W for ^1^H and ^13^C, resulting in an optimized 90° hard pulse of 9 and 6 μs, respectively, corresponding to rf efficiencies *B*_1_/√W of 3.9 and 2.9 kHz/W^½^.

### NMR spectrometer

The NMR probe is connected to a Bruker Avance III NMR spectrometer equipped with two 300-W power amplifiers for both ^1^H and ^13^C. A ^1^H band-pass filter in series with a ^13^C band stop (BS) filter, and a 32-MHz BS filter were hooked up to the ^1^H and ^13^C channels, respectively, to further minimize cross-reflection between channels.

## Supplementary Materials

### This PDF file includes:

Supplementary Text Figs. S1 to S7 Table S1 References
