# Supplementary material for: Boosting 1H and 13C NMR signals by orders of magnitude on a bench
Source: Sci Adv. 2024 Dec 4;10(49):eadq3780. doi: 10.1126/sciadv.adq3780 (PMC11616688; doi:10.1126/sciadv.adq3780)
Supplement: Supplementary file 1 — Supplementary Text Figs. S1 to S7 Table S1 References [file sciadv.adq3780_sm.pdf]

Science Advances
AAAS

Supplementary Materials for

Boosting ^{1}H and ^{13}C NMR signals by orders of magnitude on a bench

Charlotte Bocquelet *et al.*

Corresponding author: Charlotte Bocquelet, charlotte.bocquelet@univ-lyon1.fr;
Ewoud Vaneeckhaute, ewoud.vaneeckhaute@gmail.com

Sci. Adv. **10**, eadq3780 (2024)
DOI: 10.1126/sciadv.adq3780

This PDF file includes:

Supplementary Text
Figs. S1 to S7
Table S1
References

1. DNP setup

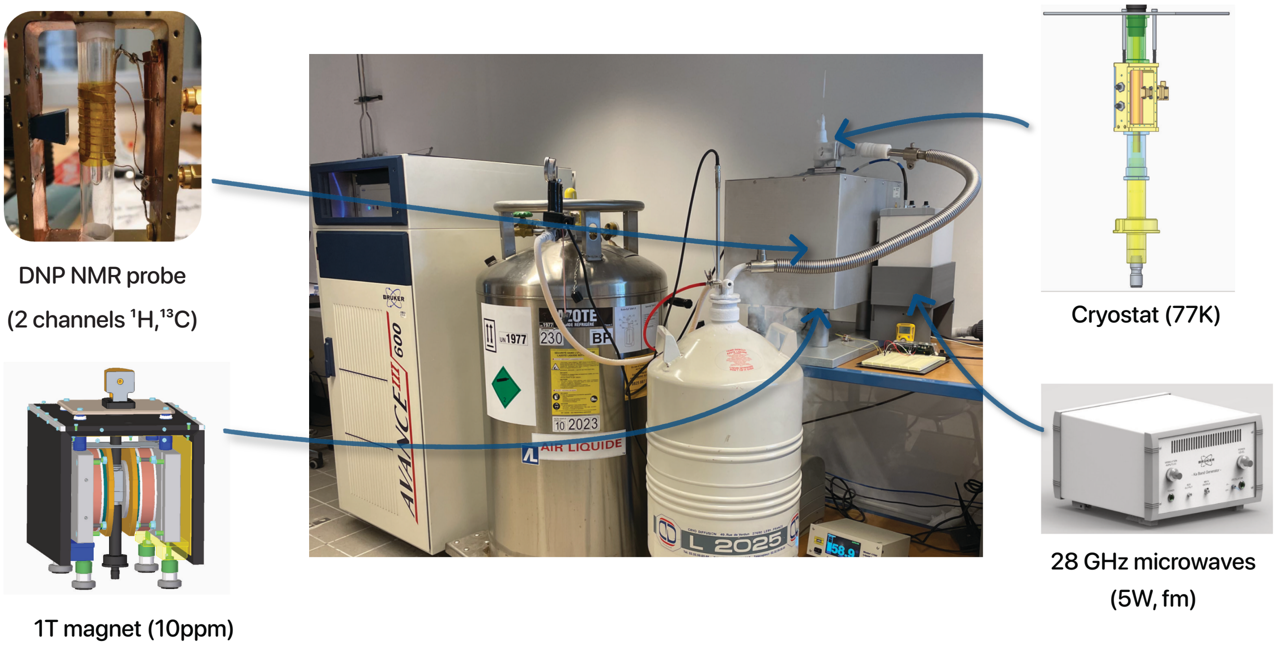

Fig. S1. Photograph of the compact DNP setup installed in our laboratory. The benchtop polarizer houses a 1T permanent magnet (bottom left) and DNP NMR probe with a copper cavity (top left). The previous version of the probe was wound around a quartz tube and then replaced by a Teflon tube as it was damaged. The microwave source (bottom right) is positioned close to the polarizer and plugged to the resonant cavity by a microwave coaxial KBL-1.5FT-LOW+ 40 GHz cable. The cryostat (top right) is inserted in the polarizer and translates into the probe insert inside the Teflon tube. The output of the cryostat, which is an opened tube, ends a few centimeters below the probe cavity. It is connected via a transfer to the heat exchanger in the smaller Dewar, supplied with a flow of g-N_2 (red tubing). The smaller Dewar is connected to a bigger one (yellow tubing) to be filled with l-N_2.

2. HYPSO-5 material properties

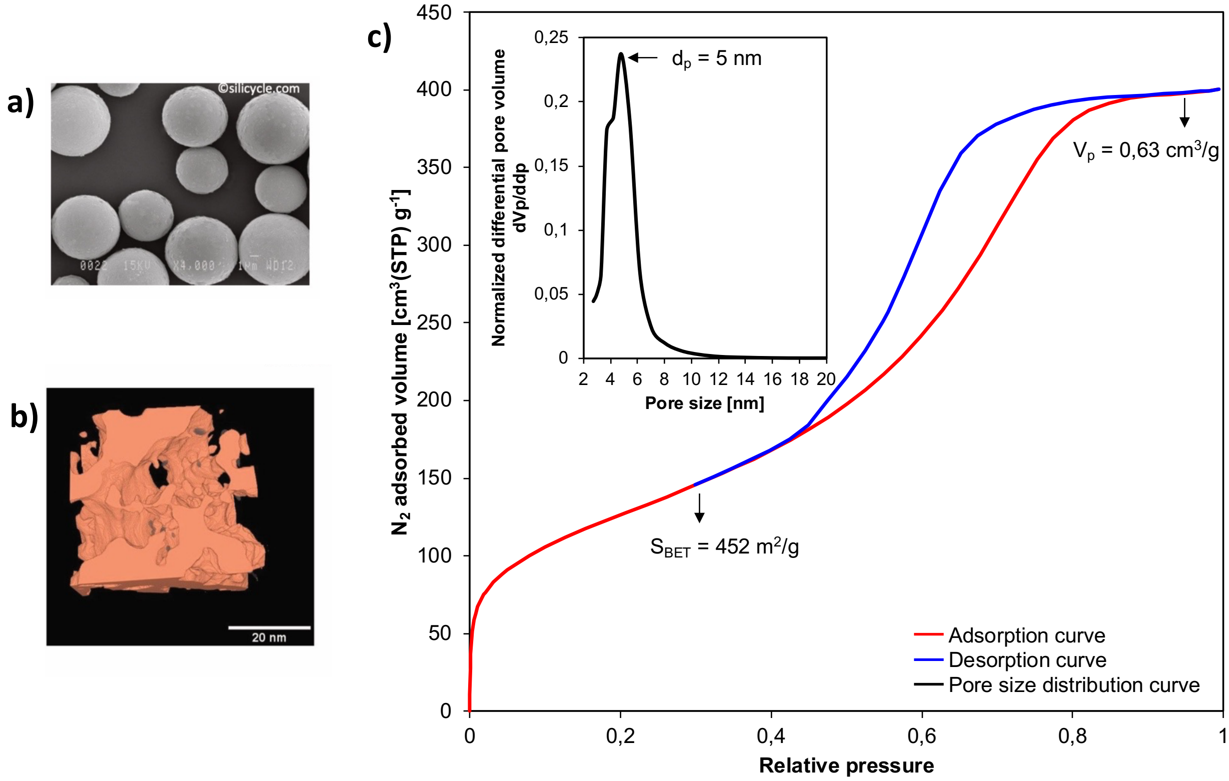

Fig. S2. Material properties of HYPSO-5. The hyperpolarizing solid used for performing DNP at 1 T and 77 K a) SEM image (provided by the supplier) of silica beads Silia*Sphere*™ exhibiting a mean particle size of 7 µm. It is noted that the silica beads used for preparing HYPSO-5 in this work have monodisperse size of 15 µm. b) Electron tomography (ET) image of HYPSO-5 material reported from (*39*) using the same porous structure c) Nitrogen adsorption-desorption isotherms at 77K and BJH pore size distribution determined from the desorption curve (inset) of the HYPSO-5 material with 43 µmol.cm^{-3} nitroxide radicals loading. HYPSO-5 powder beads have an average pore size diameter of 5 nm (70% of the pores), between 4 nm (9%) and 7 nm (21%). The detailed synthesis procedure for preparing this family of hyperpolarizing solids can be found in (*39*).

3. Magnetic field drift

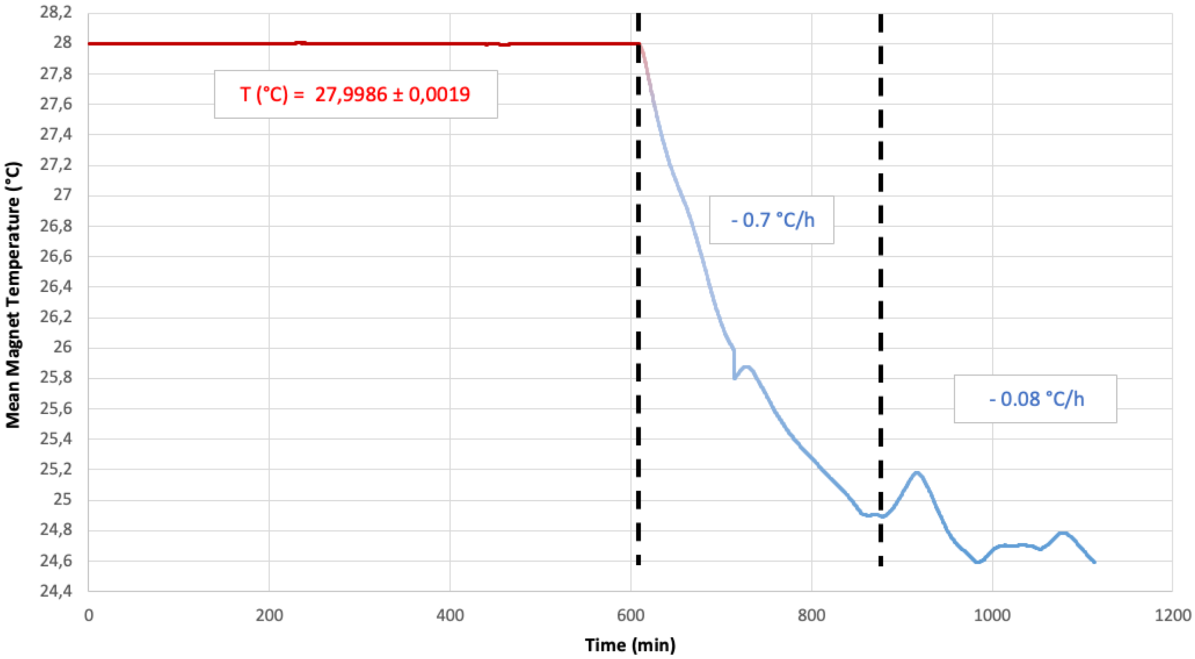

Fig. S3. Temperature monitoring inside the magnet. Monitoring of the mean temperature of the six heating plates of the magnet during a steady warm state of 10 hours and an 8-hour cold state of the cryostat at 77 K reached after a few minutes (1 min to 2 min) of cool down.

4. Microwave power and simulation

Table S1. Calibration of the microwave power by the frequency. Values of power in Watts are measured at the main output of the source and without frequency modulation.

Potentiometer position	Gate Voltage	Frequency/GHz								
		**26.6**	**27.0**	**27.5**	**27.9**	**28.0**	**28.2**	**28.4**	**28.6**	**28.8**
**0.0**	**-3.05**	0.000	0.000	0.000	0.000	0.000	0.000	0.000	0.000	0.000
**10.0**		0.001	0.012	0.003	0.000	0.000	0.000	0.000	0.000	0.000
**20.0**		0.105	0.437	0.155	0.045	0.019	0.005	0.004	0.003	0.002
**30.0**		1.023	1.862	0.891	0.468	0.263	0.117	0.100	0.091	0.063
**40.0**		0.309	3.981	2.138	1.479	1.000	0.575	0.537	0.501	0.398
**50.0**	**-2.50**	5.248	5.623	3.715	2.818	1.995	1.380	1.288	1.202	1.000
**60.0**		6.607	6.607	4.786	3.981	3.020	2.291	2.138	1.905	1.660
**70.0**		7.079	7.079	5.623	4.898	3.802	3.162	2.818	2.512	2.239
**80.0**		7.244	7.244	6.026	5.370	4.467	3.715	3.236	2.884	2.630
**90.0**		7.244	7.413	6.310	5.754	4.786	4.169	3.548	3.162	2.884
**100.0**	**-2.10**	7.244	7.413	6.457	5.888	5.129	4.467	3.802	3.311	3.020

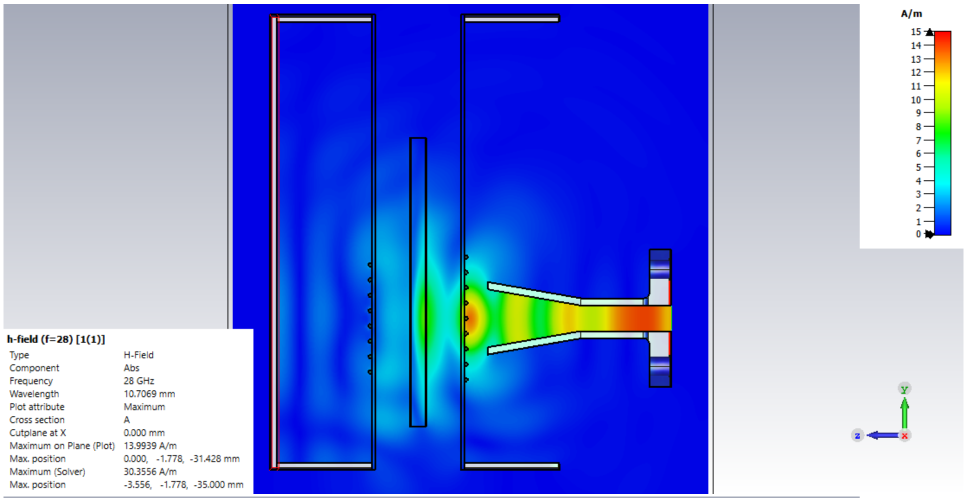
A/m
15
14
13
12
11
10
9
8
7
6
5
4
3
2
1
0
h-field (f=28) [1(1)]
Type H-Field
Component Abs
Frequency 28 GHz
Wavelength 10.7069 mm
Plot attribute Maximum
Cross section A
Cutplane at X 0.000 mm
Maximum on Plane (Plot) 13.9939 A/m
Max. position 0.000, -1.778, -31.428 mm
Maximum (Solver) 30.3556 A/m
Max. position -3.556, -1.778, -35.000 mm
y
z
x

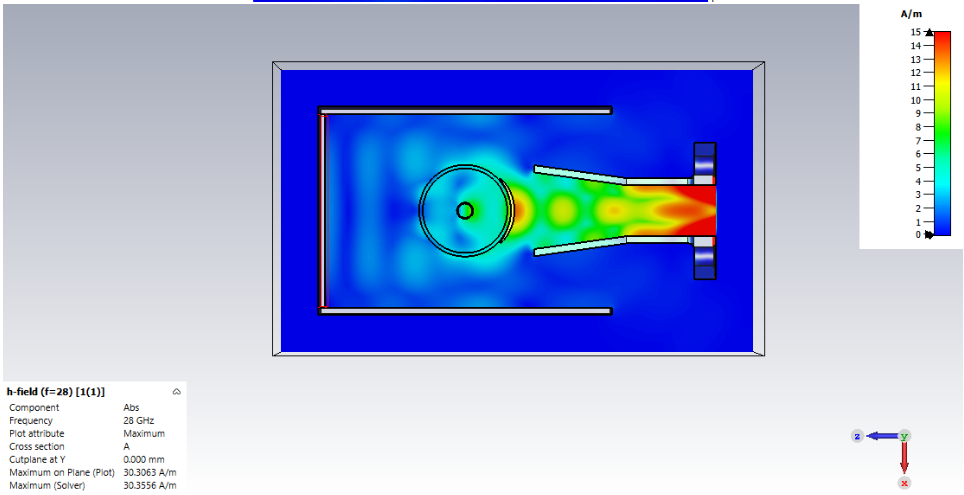
A/m
15
14
13
12
11
10
9
8
7
6
5
4
3
2
1
0
h-field (f=28) [1(1)]
Component Abs
Frequency 28 GHz
Plot attribute Maximum
Cross section A
Cutplane at Y 0.000 mm
Maximum on Plane (Plot) 30.3063 A/m
Maximum (Solver) 30.3556 A/m
z
y
x

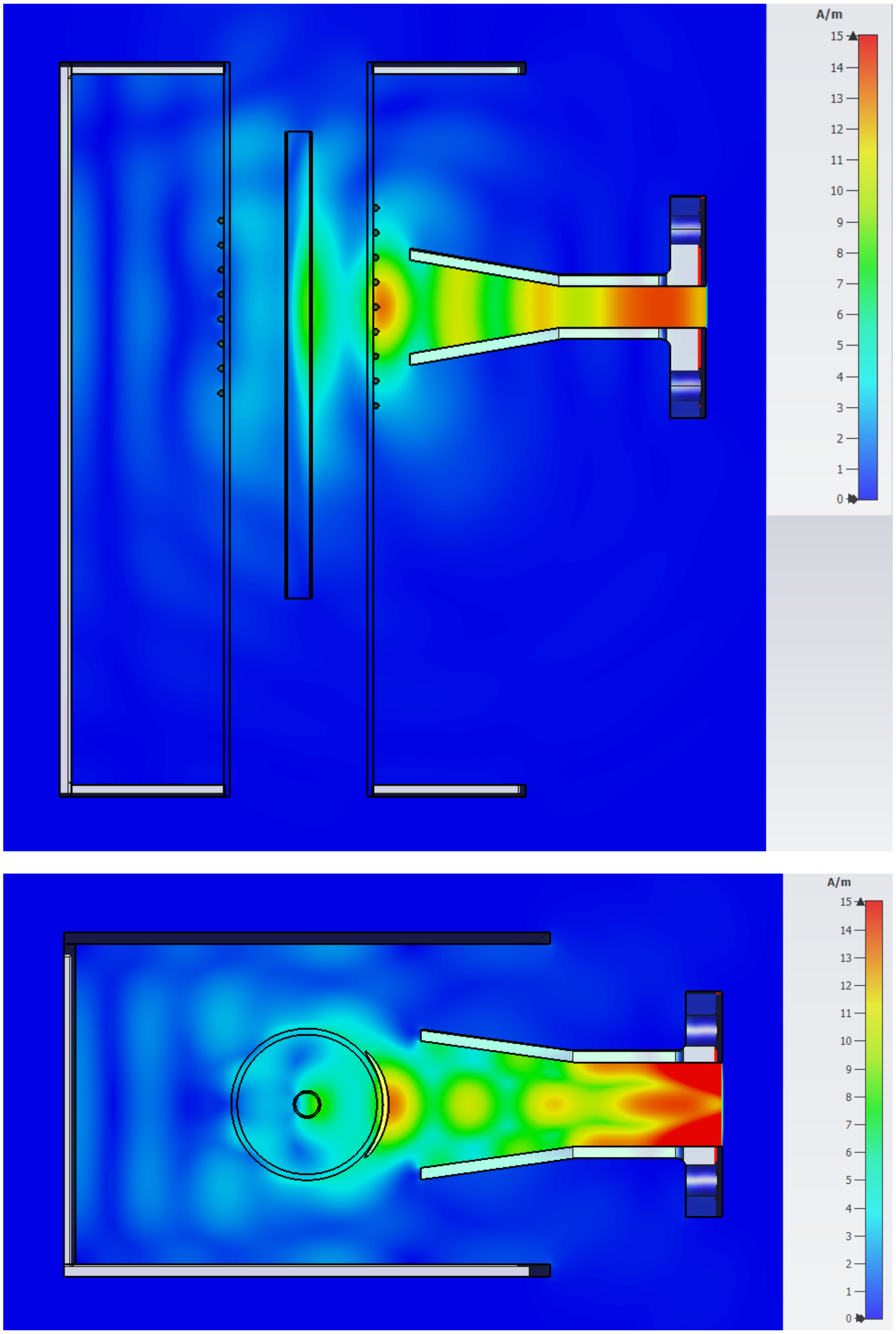

Fig. S4. Magnetic field distribution. Obtained from numerical electromagnetic field simulation using CST Microwave Studio: absolute value of H field in A/M in two orthogonal central planes is shown; the horn, coil and box have been modeled using material values for lossy copper, the coil holder and sample were modeled as dielectrics. The field was monitored at 28 GHz, following TE_{01} mode excitation at the horn flange.

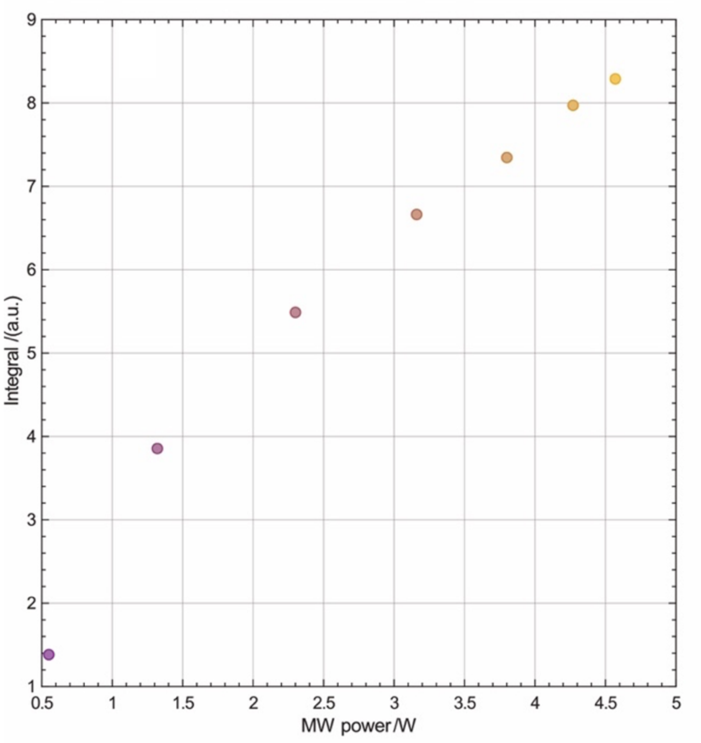

Fig. S5. DNP performance by the microwave power. Integrated ^{1}H-NMR signal during DNP in function of the microwave power at the optimal microwave frequency at 28.20 GHz for the 50 mM TEMPOL frozen solution.

5. DNP NMR Probe performances

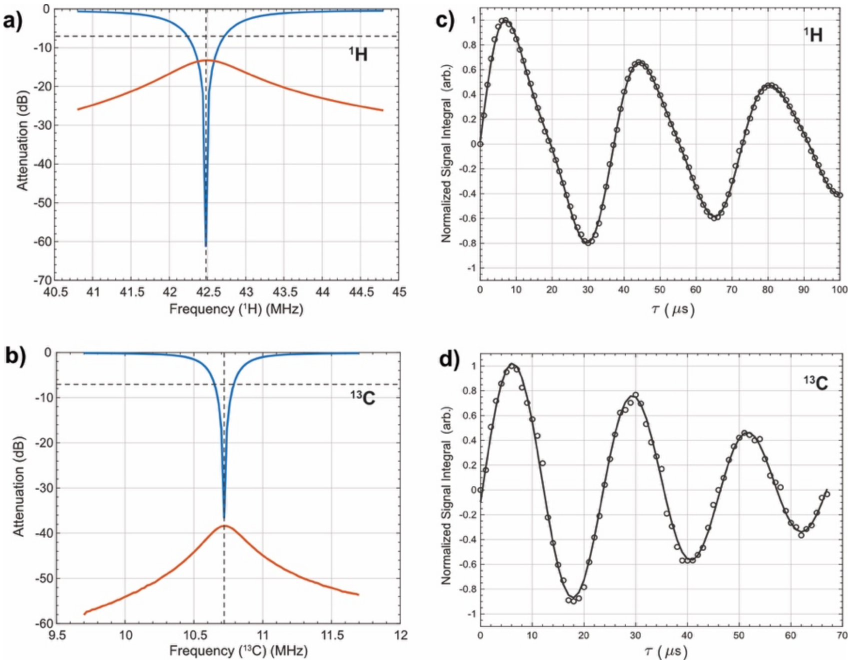

Fig. S6. VNA traces and nutation profile for both channels of the DNP NMR probe. In (**A**) and (**B**) are the vectorial network analyzer (VNA) traces of the reflectance (blue curve) and transmittance (orange curve) for the ^{1}H and ^{13}C rf coils respectively as a function of the frequency acquired at 298 K. (**C**) and (**D**) are the nutation profiles resulting from the integration of the signal acquired increasing pulse length in μs (dots), respectively on the ^{1}H and ^{13}C rf channels. The line is not a model and is simply to guide the eye. For the ^{1}H rf channel and ^{13}C rf channel respectively, the ratio of the signal integral at the 90° and 270° data points are 0.8 and 0.9. The signal attenuation is due to the incomplete excitation of the NMR linewidth of ~100 kHz.

6. Spin diffusion estimation

The pore size was chosen to allow spin diffusion to polarize all the protons of the bulk in a second in the HYPSO-5 materials. Our sample is a glass thus the diffusion tensor is isotropic and corresponds to a diffusion constant. The proton spin diffusion constant in the bulk, and in the absence of gradients due to the paramagnetic species, can be estimated from values in the literature and the same equations as in (*51*), are used here. From (*61*), a diffusion constant D = 100 nm^2.s^{-1} was found for a sample of polyethylene at 100 K in static NMR. An estimate of the spin diffusion constant of water protons impregnated in the HYPSO-5 corresponds to

$$D_2 = D_1 \left(\frac{C_2}{C_1}\right)^{\frac{1}{3}} \quad (1)$$

where D_1 is the diffusion constant of polyethylene (100 nm^2.s^{-1}) and C_1 the corresponding proton concentration (142 mol.L^{-1}). With C_2 = 22 mol.L^{-1} , the diffusion constant in our sample is D_1 = 54 nm^2.s^{-1}. We can now compute the characteristic length l over which spin diffusion would spread for a time t with (*62*)

$$l = \sqrt{4D_1 t} \quad (2)$$

Therefore, in order to be able to efficiently polarize the frozen solution within 5 seconds, the diameter of the pores shouldn't exceed 32 nm (this is neglecting the effect of nuclear spin-lattice relaxation in the radical-free frozen solution).

7. T1 rho measurements

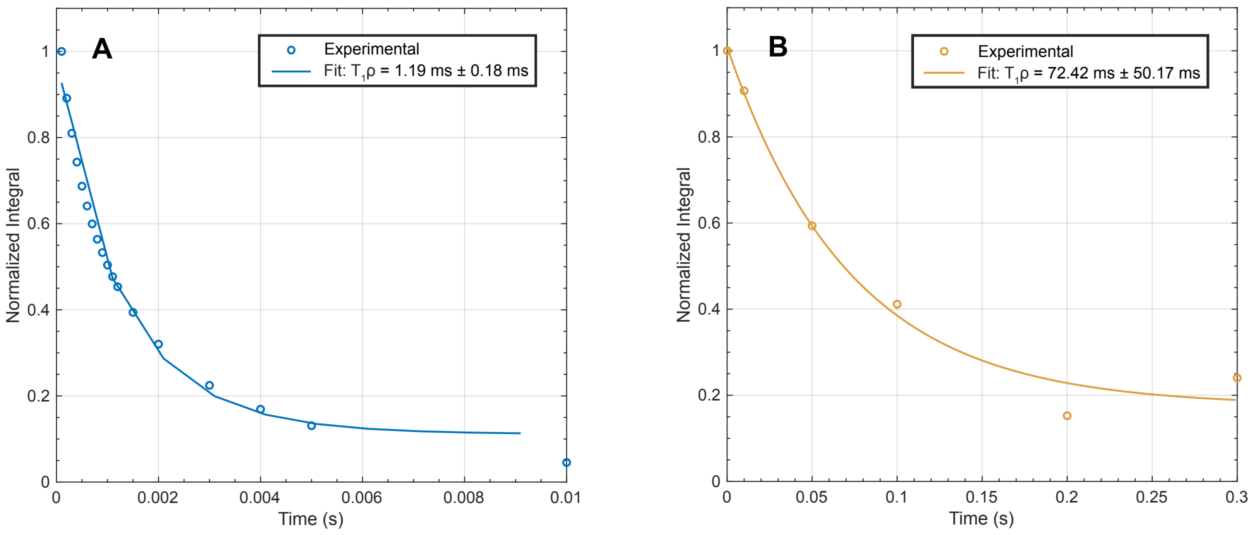

Fig. S7. T1 rho measurements. Acquired with microwave irradiation at 28.23 GHz with a triangular frequency modulation of amplitude $\Delta f = \pm$ 20 MHz at a rate f_{mod} = 60 kHz and a power $P_{\mu w}$ = 5 W. The data were fitted with a mono exponential decay I=I_0*(exp(-(t/$T_1\rho$))+d). (**A**) For proton, the spinlock duration was varied from 0.1 ms to 10 ms and for (**B**) carbon from 0.1 ms to 300 ms.

REFERENCES AND NOTES

1. J. Eills, D. Budker, S. Cavagnero, E. Y. Chekmenev, S. J. Elliott, S. Jannin, A. Lesage, J. Matysik, T. Meersmann, T. Prisner, J. A. Reimer, H. Yang, I. V. Koptyug, Spin hyperpolarization in modern magnetic resonance. *Chem. Rev.* **123**, 1417–1551 (2023).

2. J. H. Ardenkjær-Larsen, B. Fridlund, A. Gram, G. Hansson, L. Hansson, M. H. Lerche, R. Servin, M. Thaning, K. Golman, Increase in signal-to-noise ratio of > 10,000 times in liquid-state NMR. *Proc. Natl. Acad. Sci. U.S.A.* **100**, 10158–10163 (2003).

3. S. J. Elliott, Q. Stern, M. Ceillier, T. El Daraï, S. F. Cousin, O. Cala, S. Jannin, Practical dissolution dynamic nuclear polarization. *Prog. Nucl. Magn. Reson. Spectrosc.* **126-127**, 59–100 (2021).

4. S. Jannin, J.-N. Dumez, P. Giraudeau, D. Kurzbach, Application and methodology of dissolution dynamic nuclear polarization in physical, chemical and biological contexts. *J. Magn. Reson.* **305**, 41–50 (2019).

5. B. Plainchont, P. Berruyer, J.-N. Dumez, S. Jannin, P. Giraudeau, Dynamic nuclear polarization opens new perspectives for NMR spectroscopy in analytical chemistry. *Anal. Chem.* **90**, 3639–3650 (2018).

6. J.-N. Dumez, J. Milani, B. Vuichoud, A. Bornet, J. Lalande-Martin, I. Tea, M. Yon, M. Maucourt, C. Deborde, A. Moing, L. Frydman, G. Bodenhausen, S. Jannin, P. Giraudeau, Hyperpolarized NMR of plant and cancer cell extracts at natural abundance. *Analyst* **140**, 5860–5863 (2015).

7. A. Bornet, M. Maucourt, C. Deborde, D. Jacob, J. Milani, B. Vuichoud, X. Ji, J.-N. Dumez, A. Moing, G. Bodenhausen, S. Jannin, P. Giraudeau, Highly repeatable dissolution dynamic nuclear polarization for heteronuclear NMR metabolomics. *Anal. Chem.* **88**, 6179–6183 (2016).

8. A. Dey, B. Charrier, E. Martineau, C. Deborde, E. Gandriau, A. Moing, D. Jacob, D. Eshchenko, M. Schnell, R. Melzi, D. Kurzbach, M. Ceillier, Q. Chappuis, S. F. Cousin, J. G. Kempf, S.

Jannin, J.-N. Dumez, P. Giraudeau, Hyperpolarized NMR metabolomics at natural ^{13}C abundance. *Anal. Chem.* **92**, 14867–14871 (2020).

9. A. Dey, B. Charrier, K. Lemaitre, V. Ribay, D. Eshchenko, M. Schnell, R. Melzi, Q. Stern, S. F. Cousin, J. G. Kempf, S. Jannin, J.-N. Dumez, P. Giraudeau, Fine optimization of a dissolution dynamic nuclear polarization experimental setting for ^{13}C NMR of metabolic samples. *Magn. Reson.* **3**, 183–202 (2022).

10. V. Ribay, C. Praud, M. P. M. Letertre, J.-N. Dumez, P. Giraudeau, Hyperpolarized NMR metabolomics. *Curr. Opin. Chem. Biol.* **74**, 102307 (2023).

11. Q. Stern, J. Milani, B. Vuichoud, A. Bornet, A. D. Gossert, G. Bodenhausen, S. Jannin, Hyperpolarized water to study protein–ligand interactions. *J. Phys. Chem. Lett.* **6**, 1674–1678 (2015).

12. M. H. Lerche, S. Meier, P. R. Jensen, H. Baumann, B. O. Petersen, M. Karlsson, J. Ø. Duus, J. H. Ardenkjær-Larsen, Study of molecular interactions with ^{13}C DNP-NMR. *J. Magn. Reson.* **203**, 52–56 (2010).

13. Y. Kim, C. Hilty, Affinity screening using competitive binding with fluorine-19 hyperpolarized ligands. *Angew. Chem. Int. Ed. Engl.* **54**, 4941–4944 (2015).

14. Y. Kim, C. Hilty, Applications of dissolution-DNP for NMR screening. *Methods Enzymol.* **615**, 501–526 (2019).

15. K. Dos Santos, G. Bertho, C. Caradeuc, V. Baud, A. Montagne, D. Abergel, N. Giraud, M. Baudin, A toolbox for glutamine use in dissolution dynamic nuclear polarization: From enzymatic reaction monitoring to the study of cellular metabolic pathways and imaging. *ChemPhysChem* **24**, e202300151 (2023).

16. K. Luszczynski, J. A. E. Kail, J. G. Powles, Molecular motion in liquid glycerol by proton magnetic relaxation. *Proc. Phys. Soc.* **75**, 243–256 (1960).

17. N. Eshuis, R. L. E. G. Aspers, B. J. A. van Weerdenburg, M. C. Feiters, F. P. J. T. Rutjes, S. S. Wijmenga, M. Tessari, 2D NMR trace analysis by continuous hyperpolarization at high magnetic field. *Angew. Chem. Int. Ed. Engl.* **54**, 14527–14530 (2015).

18. C. R. Bowers, D. P. Weitekamp, Transformation of symmetrization order to nuclear-spin magnetization by chemical reaction and nuclear magnetic resonance. *Phys. Rev. Lett.* **57**, 2645–2648 (1986).

19. C. R. Bowers, D. P. Weitekamp, Parahydrogen and synthesis allow dramatically enhanced nuclear alignment. *J. Am. Chem. Soc.* **109**, 5541–5542 (1987).

20. M. G. Pravica, D. P. Weitekamp, Net NMR alignment by adiabatic transport of parahydrogen addition products to high magnetic field. *Chem. Phys. Lett.* **145**, 255–258 (1988).

21. R. W. Adams, J. A. Aguilar, K. D. Atkinson, M. J. Cowley, P. I. P. Elliott, S. B. Duckett, G. G. R. Green, I. G. Khazal, J. López-Serrano, D. C. Williamson, Reversible interactions with para-hydrogen enhance NMR sensitivity by polarization transfer. *Science* **323**, 1708–1711 (2009).

22. H. R. Ward. R. G. Lawler, Nuclear magnetic resonance emission and enhanced absorption in rapid organometallic reactions. *J. Am. Chem. Soc.* **89**, 5518–5519 (1967).

23. G. L. Closs, Mechanism explaining nuclear spin polarizations in radical combination reactions. *J. Am. Chem. Soc.* **91**, 4552–4554 (1969).

24. R. G. Lawler, Chemically induced dynamic nuclear polarization. *J. Am. Chem. Soc.* **89**, 5519–5521 (1967).

25. M. A. Bouchiat, T. R. Carver, C. M. Varnum, Nuclear polarization in He^3 gas induced by optical pumping and dipolar exchange. *Phys. Rev. Lett.* **5**, 373–375 (1960).

26. T. G. Walker, W. Happer, Spin-exchange optical pumping of noble-gas nuclei. *Rev. Mod. Phys.* **69**, 629–642 (1997).

27. G. R. Stadler, T. F. Segawa, M. Bütikofer, V. Decker, S. Loss, B. Czarniecki, F. Torres, R. Riek, Fragment screening and fast micromolar detection on a benchtop NMR spectrometer boosted by photoinduced hyperpolarization. *Angew. Chem. Int. Ed.* **62**, e202308692 (2023).

28. R. Kircher, J. Xu, D. A. Barskiy, *In Situ* hyperpolarization enables ^{15}N and ^{13}C benchtop NMR at natural isotopic abundance. *J. Am. Chem. Soc.* **146**, 514–520 (2024).

29. T. R. Eichhorn, A. J. Parker, F. Josten, C. Müller, J. Scheuer, J. M. Steiner, M. Gierse, J. Handwerker, M. Keim, S. Lucas, M. U. Qureshi, A. Marshall, A. Salhov, Y. Quan, J. Binder, K. D. Jahnke, P. Neumann, S. Knecht, J. W. Blanchard, M. B. Plenio, F. Jelezko, L. Emsley, C. C. Vassiliou, P. Hautle, I. Schwartz, Hyperpolarized solution-state NMR spectroscopy with optically polarized crystals. *J. Am. Chem. Soc.* **144**, 2511–2519 (2022).

30. G. R. Khutsishvili, Spin diffusion. *Sov. Phys. Usp.* **8**, 743–769 (1966).

31. L. Frydman, T. Scherf, A. Lupulescu, The acquisition of multidimensional NMR spectra within a single scan. *Proc. Natl. Acad. Sci. U.S.A.* **99**, 15858–15862 (2002).

32. L. Frydman, D. Blazina, Ultrafast two-dimensional nuclear magnetic resonance spectroscopy of hyperpolarized solutions. *Nat. Phys.* **3**, 415–419 (2007).

33. K. Singh, C. Jacquemmoz, P. Giraudeau, L. Frydman, J.-N. Dumez, Ultrafast 2D ^{1}H–^{1}H NMR spectroscopy of DNP-hyperpolarised substrates for the analysis of mixtures. *Chem. Commun.* **57**, 8035–8038 (2021).

34. C.-G. Joo, K.-N. Hu, J. A. Bryant, R. G. Griffin, In situ temperature jump high-frequency dynamic nuclear polarization experiments: Enhanced sensitivity in liquid-state NMR spectroscopy. *J. Am. Chem. Soc.* **128**, 9428–9432 (2006).

35. M. Sharma, G. Janssen, J. Leggett, A. P. M. Kentgens, P. J. M. van Bentum, Rapid-melt dynamic nuclear polarization. *J. Magn. Reson.* **258**, 40–48 (2015).

36. P. J. M. van Bentum, M. Sharma, S. G. J. van Meerten, A. P. M. Kentgens, Solid effect DNP in a Rapid-melt setup. *J. Magn. Reson.* **263**, 126–135 (2016).

37. E. R. McCarney, B. D. Armstrong, M. D. Lingwood, S. Han, Hyperpolarized water as an authentic magnetic resonance imaging contrast agent. *Proc. Natl. Acad. Sci. U.S.A.* **104**, 1754–1759 (2007).

38. R. Kircher, H. Hasse, K. Münnemann, High flow-rate benchtop NMR spectroscopy enabled by continuous overhauser DNP. *Anal. Chem.* **93**, 8897–8905 (2021).

39. O. Neudert, C. Mattea, H. W. Spiess, S. Stapf, K. Münnemann, A comparative study of 1H and 19F Overhauser DNP in fluorinated benzenes. *Phys. Chem. Chem. Phys.* **15**, 20717–20726 (2013).

40. M. Reinhard, M. Levien, M. Bennati, T. Orlando, Large ^{31}P-NMR enhancements in liquid state dynamic nuclear polarization through radical/target molecule non-covalent interaction. *Phys. Chem. Chem. Phys.* **25**, 822–828 (2022).

41. J. Phuong, Z. Romero, H. Hasse, K. Münnemann, Polarization transfer methods for quantitative analysis of flowing mixtures with benchtop ^{13}C NMR spectroscopy. *Magn. Reson. Chem.* **62**, 398–411 (2024).

42. D. Gajan, A. Bornet, B. Vuichoud, J. Milani, R. Melzi, H. A. van Kalkeren, L. Veyre, C. Thieuleux, M. P. Conley, W. R. Grüning, M. Schwarzwälder, A. Lesage, C. Copéret, G. Bodenhausen, L. Emsley, S. Jannin, Hybrid polarizing solids for pure hyperpolarized liquids through dissolution dynamic nuclear polarization. *Proc. Natl. Acad. Sci. U.S.A.* **111**, 14693–14697 (2014).

43. M. Cavaillès, A. Bornet, X. Jaurand, B. Vuichoud, D. Baudouin, M. Baudin, L. Veyre, G. Bodenhausen, J.-N. Dumez, S. Jannin, C. Copéret, C. Thieuleux, Tailored microstructured hyperpolarizing matrices for optimal magnetic resonance imaging. *Angew. Chem. Int. Ed. Engl.* **130**, 7575–7579 (2018).

44. D. Baudouin, H. A. van Kalkeren, A. Bornet, B. Vuichoud, L. Veyre, M. Cavaillès, M. Schwarzwälder, W.-C. Liao, D. Gajan, G. Bodenhausen, L. Emsley, A. Lesage, S. Jannin, C.

Copéret, C. Thieuleux, Cubic three-dimensional hybrid silica solids for nuclear hyperpolarization. *Chem. Sci.* **7**, 6846–6850 (2016).

45. T. El Daraï, S. F. Cousin, Q. Stern, M. Ceillier, J. Kempf, D. Eshchenko, R. Melzi, M. Schnell, L. Gremillard, A. Bornet, J. Milani, B. Vuichoud, O. Cala, D. Montarnal, S. Jannin, Porous functionalized polymers enable generating and transporting hyperpolarized mixtures of metabolites. *Nat. Commun.* **12**, 4695 (2021).

46. T. El Daraï, S. Jannin, Sample formulations for dissolution dynamic nuclear polarization. *Chem. Phys. Rev.* **2**, 041308 (2021).

47. K. Kundu, M. R. Cohen, A. Feintuch, D. Goldfarb, S. Vega, Experimental quantification of electron spectral-diffusion under static DNP conditions. *Phys. Chem. Chem. Phys.* **21**, 478–489 (2019).

48. S. Tanaka, Y. Nakajima, A. Ogawa, T. Kuragano, Y. Kon, M. Tamura, K. Sato, C. Copéret, DNP NMR spectroscopy enabled direct characterization of polystyrene-supported catalyst species for synthesis of glycidyl esters by transesterification. *Chem. Sci.* **13**, 4490–4497 (2022).

49. A. S. Lilly Thankamony, J. J. Wittmann, M. Kaushik, B. Corzilius, Dynamic nuclear polarization for sensitivity enhancement in modern solid-state NMR. *Prog. Nucl. Magn. Reson. Spectrosc.* **102-103**, 120–195 (2017).

50. D. Shimon, Y. Hovav, A. Feintuch, D. Goldfarb, S. Vega, Dynamic nuclear polarization in the solid state: A transition between the cross effect and the solid effect. *Phys. Chem. Chem. Phys.* **14**, 5729–5743 (2012).

51. Q. Stern, S. F. Cousin, F. Mentink-Vigier, A. C. Pinon, S. J. Elliott, O. Cala, S. Jannin, Direct observation of hyperpolarization breaking through the spin diffusion barrier. *Sci. Adv.* **7**, eabf5735 (2021).

52. M. V. Gomez, NMR reaction monitoring in flow synthesis. *Beilstein J. Org. Chem.* **13**, 285–300 (2017).

53. P. Giraudeau, F.-X. Felpin, Flow reactors integrated with in-line monitoring using benchtop NMR spectroscopy. *React. Chem. Eng.* **3**, 399–413 (2018).

54. P. Alonso-Moreno, I. Rodriguez, J. L. Izquierdo-Garcia, Benchtop NMR-based metabolomics: First steps for biomedical application. *Metabolites* **13**, 614 (2023).

55. S. Jannin, A. Bornet, S. Colombo, G. Bodenhausen, Low-temperature cross polarization in view of enhancing dissolution dynamic nuclear polarization in NMR. *Chem. Phys. Lett.* **517**, 234–236 (2011).

56. A. Bornet, R. Melzi, A. J. Perez Linde, P. Hautle, B. van den Brandt, S. Jannin, G. Bodenhausen, Boosting dissolution dynamic nuclear polarization by cross polarization. *J. Phys. Chem. Lett.* **4**, 111–114 (2013).

57. G. Menzildjian, J. Schlagnitweit, G. Casano, O. Ouari, D. Gajan, A. Lesage, Polarizing agents for efficient high field DNP solid-state NMR spectroscopy under magic-angle spinning: From design principles to formulation strategies. *Chem. Sci.* **14**, 6120–6148 (2023).

58. H. P. Erickson, Size and shape of protein molecules at the nanometer level determined by sedimentation, gel filtration, and electron microscopy. *Biol. Proced. Online* **11**, 32–51 (2009).

59. S. J. Elliott, M. Ceillier, O. Cala, Q. Stern, S. F. Cousin, S. Jannin, Simple and cost-effective cross-polarization experiments under dissolution-dynamic nuclear polarization conditions with a 3D-printed ^{1}H-^{13}C background-free radiofrequency coil. *J. Magn. Reson. Open* **10-11**, 100033 (2022).

60. E. Vaneeckhaute, C. Bocquelet, L. Bellier, H.-N. Le, N. Rougier, S. A. Jegadeesan, S. Vinod-Kumar, G. Mathies, L. Veyre, C. Thieuleux, R. Melzi, D. Banks, J. Kempf, Q. Stern, S. Jannin, Full optimization of dynamic nuclear polarization on a 1 tesla benchtop polarizer with hyperpolarizing solids. *Phys. Chem. Chem. Phys.* **26**, 22049–22061 (2024).

61. M. Roos, P. Micke, K. Saalwächter, G. Hempel, Moderate MAS enhances local ^{1}H spin exchange and spin diffusion. *J. Magn. Reson.* **260**, 28–37 (2015).

62. T. Wenckebach, “The solid effect” in *Essentials of Dynamic Nuclear Polarization* (Spindrift Publications, ed. 1, 2016), pp. 152–173.
